# Spontaneous thoracic spinal subdural hematoma associated with apixaban therapy

**DOI:** 10.1093/jscr/rjz115

**Published:** 2019-04-27

**Authors:** Javier Ardebol, Mario Cahueque, Wiliam Lopez, Enrique Azmitia

**Affiliations:** 1 Medical Research, Francisco Marroquin University, Guatemala, Guatemala; 2 Spine Center, Guatemala, Guatemala

## Abstract

Spontaneous spinal subdural hematomas are extremely rare. Most spinal hematomas are discovered in the epidural space. In the majority of cases, spontaneous hematomas are idiopathic. However, when attributed to anticoagulation therapy coumarins are more common than direct factor Xa inhibitors such as apixaban. Previous reports have linked direct factor Xa inhibitors with intracranial subdural hematomas much more frequently than spinal subdural hematomas. The manifestation of severe neurological deficits, such as sensorimotor disturbances and loss of sphincter control, is common and is considered a surgical emergency. The present case consists of a patient with a spontaneous spinal thoracic subdural hematoma secondary to apixaban use with loss of sphincter control and paraplegia. After 6 months of follow-up, the patient recovered completely.

## INTRODUCTION

Spinal subdural hematomas (SSDHs) are quite rare, as they appear much less frequently than intracranial subdural hematomas and spinal epidural hematomas. Subdural spinal hematomas represent ~4.1% of all intraspinal hematomas [1]. The three primary causes of SSDHs are post-traumatic, iatrogenic and spontaneous [2]. In many cases, neurological symptoms are evident, therefore establishing an immediate diagnosis utilizing an imaging modality, such as magnetic resonance, is compulsory. Once the diagnosis has been confirmed, correlation with clinical severity is necessary to justify whether surgical intervention is required [1, 3]. The following report consists of a rare case of a spontaneous thoracic subdural hematoma associated with paraplegia and sphincter dysfunction secondary to apixaban use.

## CASE REPORT

A 67-year-old female patient who comes to emergencies for presenting lower extremity paraplegia She had a 3-year history of atrial fibrillation on treatment with apixaban 5 mg per day.

**Figure 1:**
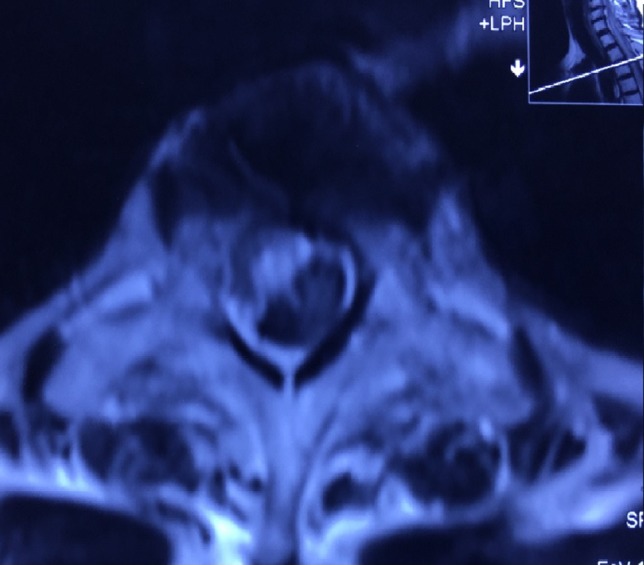
MRI shows subdural hematoma T4 to T7.

**Figure 2:**
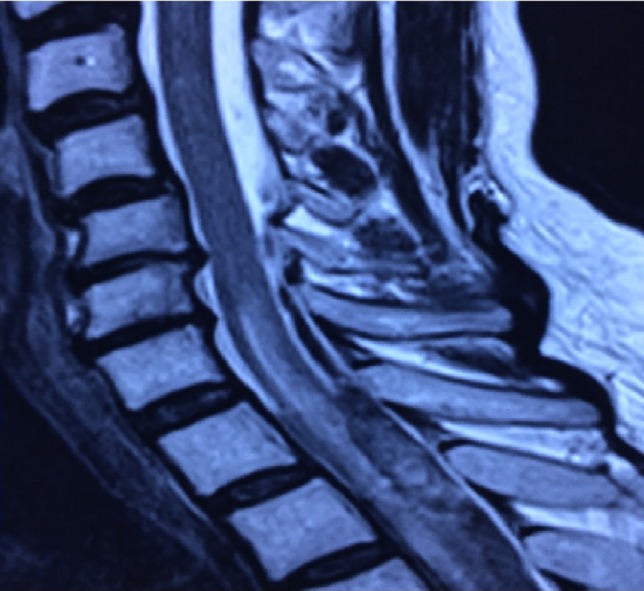
MRI shows subdural hematoma T4 to T7.

Patient presented sudden onset with dorsal pain followed immediately by bilateral lower extremity paresis that progressed to complete paraplegia with bowel and bladder dysfunction over 15 min. The patient was taken to a local hospital where an MRI was performed that demonstrated a SSDH extending from T4 to T7 with some intramedullary enhancement noted (Figs 1 and 2). Loss of sensory level from T10 up to ~T8 level. She denied upper extremity complaints.

**Figure 3:**
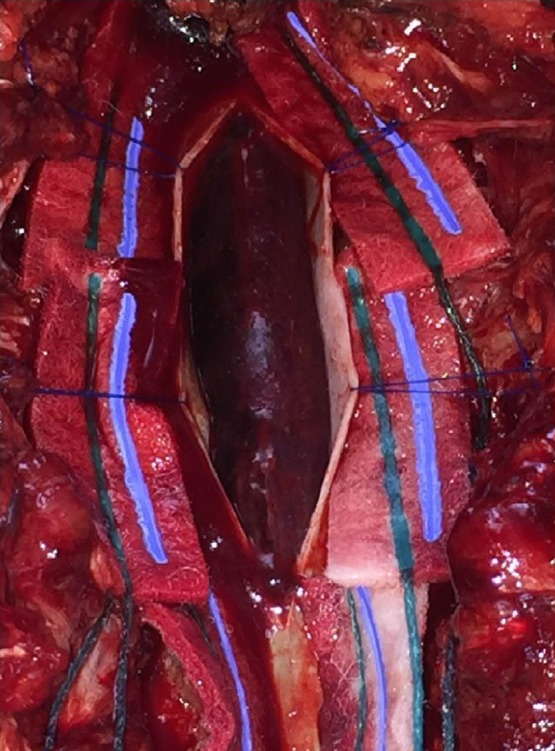
Presence of subdural hematoma after durotomy.

**Figure 4:**
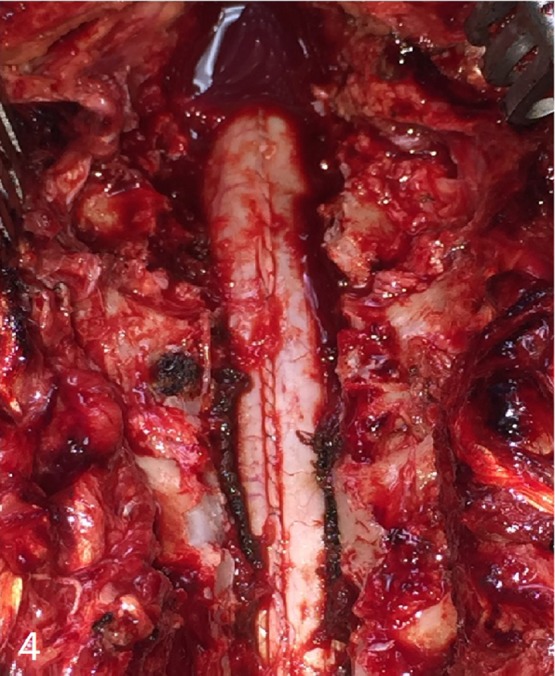
Image after drainage of the hematoma.

Patient is taken to the operating room immediately, where a wide laminectomy was performed from T4 to T7, durotomy and drainage of subdural hematoma (Figs 3 and 4). No complications during surgery.

The 24 h post-operative patient persists with bowel and bladder dysfunction, mobilizes lower extremities (muscle strength 2/5). She initiates physical therapy and rehablitation.

Three months post-operative, full recovery of muscle strength, but still persists with bladder problems, but since they are mild.

One year post-operative, full recovery, she used for 4 months posture corrector (brace) for risk of kyphosis, control radiographs do not show increased thoracic kyphosis. Patient with complete satisfaction.

## DISCUSSION

Spinal hematomas are discovered more frequently in the epidural space. Hence, SSDH are infrequently reported. Spontaneous SSDHs are associated with factors such as neoplasms, vascular malformations and coagulopathies, either hereditary or drug-induced. The majority of SSDHs secondary to anticoagulant therapy are attributed to coumarin use [1–3].

SSDHs occur predominantly in the thoracic region [1, 4]. There is uncertainty regarding the exact mechanism, although one theory has been proposed. Unlike the spinal epidural space or the intracranial subdural space, there are no major blood vessels or bridging veins in the spinal subdural space. However, this theory suggests that when intrathoracic pressure rises significantly, hemorrhage occurs in the more vascular subarachnoid space and infiltrates through the arachnoid mater into the subdural space where blood accumulation occurs [5].

The usual clinical scenario consists of back pain that radiates to the upper or lower extremities along with either sensorimotor disturbances or autonomic dysfunction, or both [4, 6]. The most common clinical manifestation is progressive motor weakness. Although, approximately half of patients will refer an intense spear-like pain at the site of the hematoma. Symptom appearance varies from hours to weeks, yet prompt recognition is advised to avoid irreversible neurological deficits [1, 4, 6].

Magnetic resonance remains the imaging modality of choice for the diagnosis of spinal hematomas [4, 7]. SSDHs are less probable to occur in the upper thoracic region rather than the lower thoracic region. Unequivocally, hematomas are described as subdural if there is no displacement of the dura mater. Spinal hematomas are classified into phases according to the relation between symptom onset and MRI realization.

- Hyperacute (within 12 h of symptom appearance).
- Acute (12 h to 3 days).
- Subacute (3 days to 2 weeks).
- Chronic (2 weeks to years).

When the subdural hematoma is discovered during the hyperacute phase, such as with the patient described in the case report, the blood collection is visualized as isointense and hyperintense in T1 and T2 signals, respectively. The intensity will normally vary according to the phase in which the MRI has been performed [4, 7, 8]. Once the presence and location of the subdural hemorrhage are determined it is fundamental to decide the optimal treatment for the patient unhesitantly.

Decompressive laminectomy and surgical drainage of the hemorrhage is the treatment of choice [1, 3, 9]. Prompt surgical intervention is necessary to avoid a permanent neurological deficit. Spontaneous resolution of intraspinal hematomas is rarely documented. Conservative treatment is a viable option when neurological symptom severity is grade D or E in the Frankel Scale. Surgeon criterion is advised on every individual case as surgical intervention augments the risk of iatrogenic spinal cord injury and excessive bleeding in those under anticoagulant therapy [8, 10]. Nonetheless, operative treatment is a necessary risk when a severe neurological deficit is evident and, more importantly, corrigible.

## CONCLUSION

In this particular case, the patient presented with an unusual spontaneous upper thoracic SSDH. The clinical presentation consisted of serious neurological symptoms. Urgent surgical treatment was performed, and hematoma drainage was successful. The patient completely recovered after 6 months of follow-up. The patient spontaneously developed the hematoma secondary to the use of apixaban which is rare since major hemorrhage is more commonly linked to rivaroxaban. Clinicians are advised to consider this neurological emergency in patients under anticoagulation therapy, regardless of presumed drug safety, to prevent permanent deleterious effects.
